# A review on federated learning in computational pathology

**DOI:** 10.1016/j.csbj.2024.10.037

**Published:** 2024-10-29

**Authors:** Lydia A. Schoenpflug, Yao Nie, Fahime Sheikhzadeh, Viktor H. Koelzer

**Affiliations:** a Department of Pathology and Molecular Pathology, University Hospital and University of Zürich, Zürich, Switzerland; b Roche Diagnostics, Digital Pathology, Santa Clara, CA, United States; c Institute of Medical Genetics and Pathology, University Hospital Basel, Basel, Switzerland; d Department of Oncology, University of Oxford, Oxford, UK; e Nuffield Department of Medicine, University of Oxford, Oxford, UK

**Keywords:** Pathology, Federated learning, Privacy preservation, Artificial intelligence, Medical informatics

## Abstract

Training generalizable computational pathology (CPATH) algorithms is heavily dependent on large-scale, multi-institutional data. Simultaneously, healthcare data underlies strict data privacy rules, hindering the creation of large datasets. Federated Learning (FL) is a paradigm addressing this dilemma, by allowing separate institutions to collaborate in a training process while keeping each institution's data private and exchanging model parameters instead. In this study, we identify and review key developments of FL for CPATH applications. We consider 15 studies, thereby evaluating the current status of exploring and adapting this emerging technology for CPATH applications. Proof-of-concept studies have been conducted across a wide range of CPATH use cases, showcasing the performance equivalency of models trained in a federated compared to a centralized manner. Six studies focus on model aggregation or model alignment methods reporting minor (0∼3%) performance improvement compared to conventional FL techniques, while four studies explore domain alignment methods, resulting in more significant performance improvements (4∼20%). To further reduce the privacy risk posed by sharing model parameters, four studies investigated the use of privacy preservation methods, where all methods demonstrated equivalent or slightly degraded performance (0.2∼6% lower). To facilitate broader, real-world environment adoption, it is imperative to establish guidelines for the setup and deployment of FL infrastructure, alongside the promotion of standardized software frameworks. These steps are crucial to 1) further democratize CPATH research by allowing smaller institutions to pool data and computational resources 2) investigating rare diseases, 3) conducting multi-institutional studies, and 4) allowing rapid prototyping on private data.

## Graphical abstract

**Figure:**
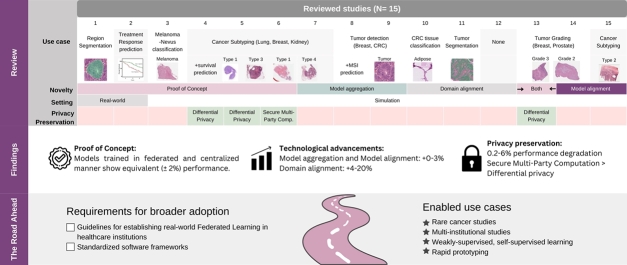

## 1 Introduction

The advancement of machine learning (ML), and particularly deep-learning (DL) necessitates access to large-scale, multi-institutional data. This is a non-trivial problem for development of AI solutions in healthcare, where data access is carefully restricted to preserve patient privacy. Federated Learning (FL) emerges as a paradigm addressing this dilemma by enabling geographically separate institutions to collaborate in a model training process, without requiring data to leave the institution of origin. Each institution utilizes their data and hardware resources for training and iteratively shares their trained local model for aggregation into a global model, combining insights from all participating institutions. Such a collaborative approach is particularly relevant to the field of pathology, where an increasing digitization of tissue specimens in the form of whole slide images (WSIs) is currently leading to a massive influx in available data, warranting the need for data-based, but also privacy preserving, analysis methods [1], [2]. Computational techniques for the analysis of pathological data are referred to as computational pathology (CPATH) algorithms, including cell-level tasks such as cell detection and classification [3], [4], mitosis detection [5], [6], region-level tasks such as tissue segmentation [7], as well as slide-level prediction of cancer subtype or grade [8], [9], [10], molecular alterations [11], [12], or recurrence risk [13], [14]. In this paper we introduce core principles of FL and provide an in-depth review of key developments of FL in CPATH. We provide a perspective on the road ahead to stimulate further research and discussion in the field, highlighting both opportunities and challenges to unlocking the potential of FL for computational pathology.

## 2 Basic principles of federated learning

This section introduces the basic principles of FL, from its operational workflow to challenges and considerations when applying FL.

### 2.1 Operational workflow of FL

**Fig. 1:**
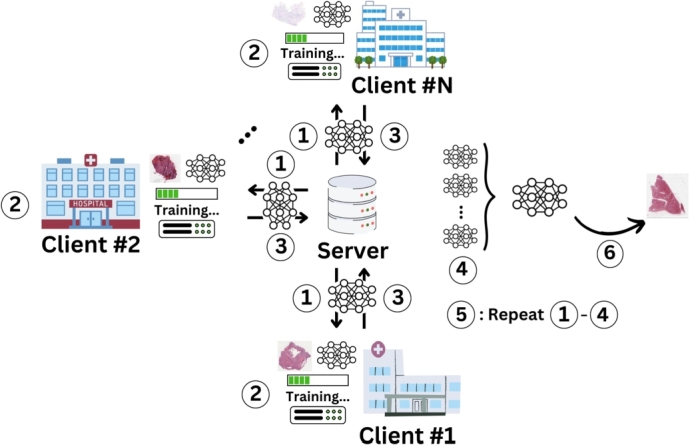
Operational FL workflow visualized for a horizontal FL use case for pathology: (1) Initialization, (2) Local Training, (3) Model Update Transmission, (4) Aggregation, (5) Iteration: Repeat (1)-(4) until convergence criteria is reached, (6): Deployment.

FL addresses data privacy and security concerns by enabling collaborative model training without the need for centralized data storage. In FL, the training data remains distributed across multiple devices or nodes, referred to as “clients”, which are commonly located at separate institutions. Each client trains a local model on its data and only the model updates, rather than the raw data, are sent to a central server. The central server then aggregates these updates to form a global model. This iterative process continues until the global model converges to an optimal performance. FL can be differentiated into vertical and horizontal FL, where vertical FL refers to clients having datasets of the same modality and horizontal to differing data modalities across clients. Fig. 1 demonstrates the operational workflow of FL, shown for a single-modality, horizontal setup, which can be summarized in the following steps (1) Initialization, (2) Local Training, (3) Model Update Transmission, (4) Aggregation, (5) Iteration and (6) Deployment. First, the central server initializes a global model and distributes it to all participating clients. This initial model can be pre-trained on a public dataset or randomly initialized. Second, each client trains the received global model on its local dataset. During this local training phase, the model learns to adapt to the local data distribution, capturing unique patterns and features. Third, after local training, each client computes and transmits the updates of the model parameters to the server. These updates typically include gradients or weights that reflect the learning adjustments made during the local training phase. Fourth, the central server collects the model updates from all participating clients and aggregates them to update the global model. Fifth, the updated global model is then redistributed to the clients, and the process iterates. Each iteration is referred to as a communication round. The process continues until the global model achieves convergence. Lastly, once the global model has converged, it is deployed for inference. The global model, now trained on diverse and distributed data, is expected to generalize well to new data. Please refer to [15] for further reading on Federated Learning principles.

### 2.2 Challenges and considerations

While FL offers significant advantages in terms of privacy and security, there are also several intrinsic challenges that need to be addressed [16], [17]. First, data heterogeneity, as clients may have non-independent and identically distributed (non-IID) data, which can lead to discrepancies in the local models and affect the convergence of the global model. Second, privacy leaks, while data is kept private and not shared during FL, the shared model parameters contain information about the data, making it vulnerable to privacy attacks, therefore requiring further consideration of privacy. Privacy preservation techniques address and prevent such attacks, but simultaneously introduce computational overhead and affect model accuracy [18], [19]. Finding an optimal trade-off between privacy, efficiency and performance is an ongoing research challenge. Third, communication overhead, FL involves frequent communication between clients and the central server, leading to potential network congestion and high communication costs. Fourth, system heterogeneity, clients may have varying computational capabilities and network conditions; this leads to asynchronous updates and potential delays. Lastly, scalability, as the management of communication and computation becomes more complex with an increasing number of participating clients. However, this is less critical in pathology applications, where the number of clients is typically in the 2-100 range, in contrast to consumer FL targeting millions of mobile devices as potential clients.

## 3 Study design

**Fig. 2:**
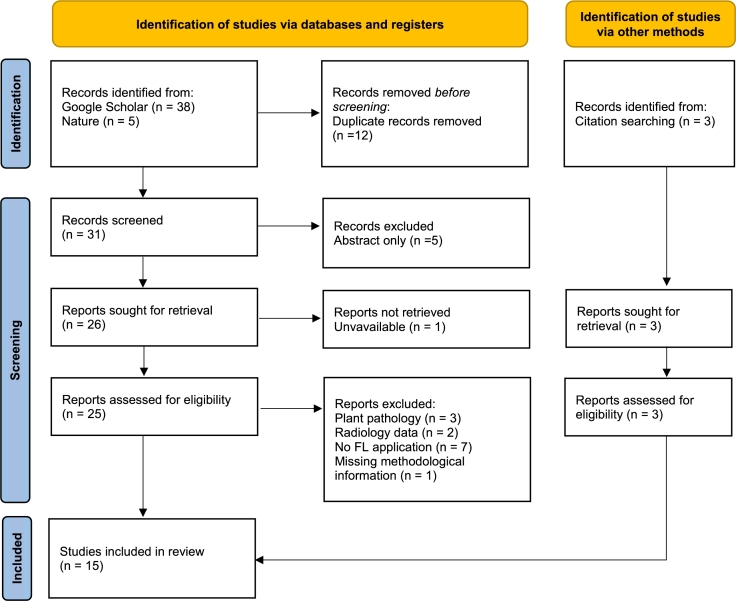
Flowchart showing the literature search and systematic review process according to the Preferred Reporting Items for Systematic Reviews and Meta-Analyses (PRISMA) recommendations [20].

This systematic review aims to answer the research question: What are the key developments and finding of FL applications in the field of CPATH? Studies were identified in July 2024 by online search on Google Scholar for publications containing the following keyword combinations in their title: “federated” + “pathology” (N=19), “federated learning” + “histopathology” (N=10), “federated learning” + “histology” (N=3), “federated learning” + “whole slide images” (N=6). We further searched for publications in Nature journals containing the keywords “federated learning” and “histopathology”, with restrictions to research articles and the subjects cancer, computational biology and bioinformatics, health care, medical research and oncology resulting in N=5 matches. Leading to a total of N=43 publications (Fig. 2), published within the timeframe of August 2021- July 2024, originating from. Before in-depth screening we removed 12 duplicate results, after screening we further excluded 5 publications for which only an abstract was available. On retrieval a report was excluded due to unavailability, resulting in n=25 reports assessed for eligibility. Based on our research question, we solely consider records applying FL methods for computational pathology and restrict the data type to mammal derived pathology data. This lead to exclusion of 7 records, which did not apply FL methods, 3 investigating plant pathologies, 2 considering radiology data, and one further exclusion due to missing methodological information, resulting in a total of 12 considered studies. During paper review we identified 3 additional publications to be included in the review, leading to a final number of 15 publications included within this review.

## 4 Key techniques and developments of federated learning for pathology

In this section, we will focus on the specific progress of FL within the CPATH domain. We first introduce the unique challenges of CPATH to enable a deeper understanding of the field. We then provide a survey of studies, including proof of concepts (PoCs) demonstrating the applicability of FL to various CPATH tasks, as well as technological advances of FL methods tailored to meet the distinct challenges and needs of CPATH. Based on the study selection, inclusion and exclusion criteria detailed in the previous section, we consider 15 studies (Table 1) for in-depth review.

**Table 1:** Overview of studies applying FL to CPATH tasks. Novel aggregation methods are marked by “*”. BRCA: Breast Cancer Gene, CCRCC: Clear Cell Renal Cell Carcinoma, CRC: Colorectal Cancer, IFTA: Interstitial Fibrosis and Tubular Atrophy, IDC: Invasive Ductal Carcinoma, MSI: Microsatellite Instability, RCC: Renal Cell Carcinoma, SMC: Secure Multi-Party Computation, DP: Differential Privacy, TNBC: Triple-Negative Breast Cancer.

Ref.	Novelty	Label type	Task	Aggregation	Setting, # clients	Evaluation	
						Test sets	Baseline algorithms
[34]	PoC: Real-world deployment	Pixel	IFTA and glomeruli segmentation	FedAvg	Real-world, N=6	Held-out, External	Centralized, Local
[44]	PoC: Rare cancer	Slide	Treatment response prediction in TNBC	FedAvg	Real-world, N=4	Held-out, External	Local, FedAvg, Ensemble, SCAFFOLD [45]
[40]	PoC: WSI classification	Slide	Melanoma-nevus classification	FedAvg	Simulation, N=5	Held-out, External	Centralised, Ensemble
[41]	PoC: MIL	Slide	BRCA+RCC subtyping, CCRCC survival prediction	Weighted FedAvg	Simulation, N=2	Held-out	Centralized, Local
[42]	PoC: Differential privacy	Slide	Lung cancer subtyping	FedAvg	Simulation, N=[4,8,16,32]	Held-out	Centralised, Local, FedAvg
[43]	PoC: SMC	Slide	Lung cancer subtyping	FedAvg	Simulation, N=6	Held-out	FedAvg+DP
[33]	Aggregation algorithm	Slide	Lung and kidney cancer subtyping	Prop-FFL*	Simulation, N=[4,6]	Held-out	FedSGD [46], q-FedSGD [47]
[32]	Aggregation algorithm	Tile, Slide	CRC detection, MSI prediction	FedDropout-Avg*	Simulation, N=11	Held-out, External	Local, FedAvg, FedProx [48], FedBN [49], PF L [50]
[31]	Aggregation algorithm	Tile	Breast tumor detection	SiloBN*	Simulation, N=[2,5]	Held-out	Centralized, Local, FedAvg
[36]	Domain alignment	Tile, Slide	Stain normalization, Colorectal tissue classification, Breast cancer subtyping	FedAvg	Simulation, N=5-8	Held-out	LG-FedAvg [51], FedPer [52], FedBN [49]
[37]	Domain alignment	Pixel	Stain normalization, prostate tumor segmentation	FedAvg	Simulation, N=20	Held-out	FedAvgM [53][53]
[35]	Domain alignment	None	Stain normalization	FedAvg	Simulation, N=8	Held-out	Stain normalization methods
[38]	Model and Domain alignment	Slide	Breast IDC grading	FedAvg	Simulation, N=3	Held-out	Local, FedAvg, FedProx [48], FedBN [49], MOON [54]
[39]	Model alignment	Slide	Prostate tumor detection, Gleason grading	Weighted FedAvg	Simulation, N=[6,7]	Held-out, External	Centralized, Local, FedAvg
[55]	Multi-modality, Model alignment	Slide	Multi-modal lung and kidney cancer subtyping	FedAvg	Simulation, N=3	Cross-validation	Local, Multi FedAvg [55]

### 4.1 Unique challenges of CPATH

**Fig. 3:**
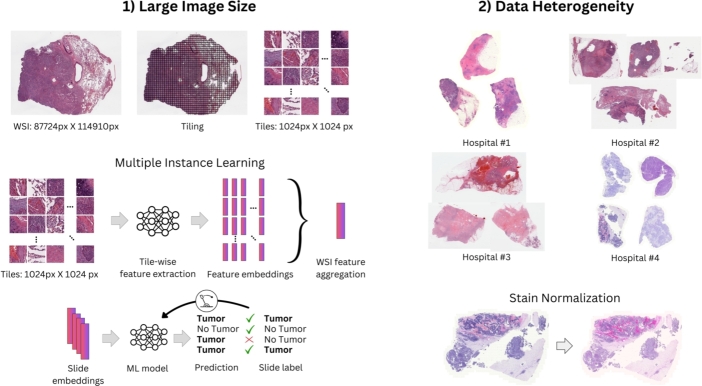
Challenges in CPATH: 1) The gigapixel size of WSIs necessitates tiling and tile-wise application of DL models. To allow tasks based on slide-level labels, MIL aggregates tile-level features into a slide-level feature embedding, on which a lightweight ML model can be trained. 2) Differing tissue preparation, staining and scanners across institutions lead to data heterogeneity. One possible method of overcoming pre-analytical variability is stain normalization.

Pathology encompasses the study and diagnosis of diseases through the examination of tissues, cells, and bodily fluid. Digital pathology, which involves the acquisition, management, sharing, and interpretation of pathology information in a digital environment, is the backbone of clinical development and application of CPATH [21]. Understanding the unique challenges of CPATH requires an understanding of how routine tissue sections are prepared and digitized in the pathology laboratory: Patient tissue samples are first fixed in formalin and embedded in paraffin for preservation, sectioned at 3−5μm, placed on glass slides, and stained to highlight different cellular components. The slides are then scanned to create high-resolution digital whole slide images (WSIs), with a resolution commonly in the range of 0.25-1 microns per pixel (MPP), e.g. a glass slide of size 75 mm x 25 mm results in an WSI of 150,000px x 50,000px for 0.5 MPP at 20x magnification. The first challenge arises due to such large image size, which necessitates tile-based processing for high-resolution WSI analysis (Fig. 3).

Labels for tile-level tasks require expert annotations which are labor intensive. To allow training a CPATH algorithm with less expensive slide-level labels, techniques such as Multiple Instance Learning (MIL) [22], [23] employ tile-level feature extraction, followed by aggregation into slide-level representations and then predictions. A second challenge in CPATH arises due to the variation of pre-analytical conditions across different labs. Different tissue sample preparation processes, staining procedures and whole-slide scanners can result in varying color and intensity of the digitized slides, leading to significant data heterogeneity. This has been addressed by incorporating multi-institutional data in the algorithm development [5], or through techniques such as stain normalization (Fig. 3) [24], [25], stain augmentation [26] or domain adversarial training [27]; as well as utilizing models which have been pre-trained using self-supervised learning (SSL) on large-scale, multi-institutional datasets [28], [29], [30]. While the first challenge of large image size is independent of the training setting (centralized or federated), the second challenge of data heterogeneity originates from the training data and is therefore directly impacted by a federated setting where data is distributed across different clients.

### 4.2 Proof of concepts

Numerous studies have been conducted to demonstrate the PoCs of FL in CPATH. These studies cover a wide range of cancer types and tissue image analysis tasks, showing the applicability of FL in the CPATH domain. Studies focusing on tile binary classification (benign vs. malignant) tasks have been conducted for breast [31] and colorectal cancer [32]. Tile multi-class classification has been investigated for kidney and lung cancer subtyping [33]. Region segmentation is explored in [34] to segment interstitial fibrosis and tubular atrophy (IFTA), as well as glomeruli in renal tissue biopsy. To address the unique challenges of stain variation in CPATH, generative adversarial network (GAN)-based stain normalization [35], [36], [37] and pseudo-image generation [38] are investigated in the FL setting. At the slide-level, various clinical applications have been explored, which include prostate cancer diagnosis and Gleason grading [39], melanoma diagnosis [40], cancer subtyping [41], [42], [43], microsatellite instability (MSI) prediction [32], patient survival prediction [41], and treatment response prediction [44] (Table 1). The majority of the slide-level tasks are tackled using the weakly supervised MIL technique, while [40], [32] are essentially tile classification with simple slide-level aggregation such as average and max pooling.

For FL model evaluation, all studies report performance on a held-out test set from the same source as the training data. Some further validate the FL model on an external test set [40], [42], [34], [32], [44], [39]. The comparison baselines can be categorized into centralized models (trained on all available data in the central location), local models (trained on data from a single client), ensemble models (ensemble of all local models), as well as FL models utilizing different aggregation settings (Table 1).

The following findings are noteworthy. First, FL models are generally reported to perform on par (±2%) or slightly worse (2∼6% lower) than centralized models across all studies, demonstrating the feasibility of achieving performance equivalency, yet also highlighting the more challenging optimization space in FL. Second, in [44], [41], [32], [34], [39] FL models are shown to generalize better across all client's held-out test sets than each client's local model. Similar observations are made in studies considering local and/or centralized model performance on external test sets [44], [40], [42], [34], [39], where the FL model performs better (>+2%) than or similar (±2%) to all local models. This indicates an overall improved domain generalizability of the FL model compared to local models. Third, most ensemble models surprisingly show similar (±2%) or better (>+2%) performance compared to FL models on both held-out and external datasets in [40], [44]. Ensemble models can be viewed as an extreme case of FL, where only a one-time model aggregation step is performed at the inference phase. This requires deploying all local models and entails a higher inference computational cost proportional to the number of clients. Nonetheless, the comparable performance of the ensemble and FL models motivates further investigations. Lastly, we note that the majority of studies, excluding [34] and [44], were conducted in a simulated environment, where clients are not located at physically separate institutions, showing the challenges of implementing real-world FL systems.

### 4.3 Technological advances

We consider technological advances in applying FL to CPATH by examining the challenges they aim to address. As previously identified, data heterogeneity and privacy preservation are the main challenges in both CPATH and FL. Several methodologies have been proposed to address and mitigate these challenges (Table 2). These methodologies can be categorized into model aggregation, model alignment, domain alignment, and privacy preservation methods.

**Table 2:** Technological advancements proposed for FL application in pathology to achieve a better consideration of data heterogeneity and improved data privacy through novel aggregation methods, domain alignment methods and privacy preservation techniques. N/A indicates no specific method was applied or the method is unknown.

Ref.	Aggregation	Model alignment	Privacy preservation	Domain alignment	Addressed challenge(s)
[31]	SiloBN	N/A	N/A	N/A	Data heterogeneity, Privacy preservation
[32]	FedDropout-Avg	N/A	N/A	N/A	Data heterogeneity, Privacy preservation
[33]	Prop-FFL	N/A	N/A	N/A	cell
[39]	Weighted FedAvg	FACL	DP	N/A	Data heterogeneity
[41]	Weighted FedAvg	N/A	DP	N/A	Data heterogeneity, Privacy preservation
[55]	FedAvg	FedMM	N/A	N/A	Data heterogeneity, Multi-modality
[43]	FedAvg	N/A	SMC	N/A	Privacy preservation
[42]	N/A	N/A	DP	N/A	Privacy preservation
[35]	FedAvgM	N/A	N/A	Stain normalization GAN	Data heterogeneity
[36]	FedAvg	N/A	N/A	Stain normalization GAN	Data heterogeneity
[37]	FedAvg	N/A	N/A	Stain normalization with BottleGAN	Data heterogeneity
[38]	FedAvg	FL-BT	N/A	SSL on GAN-generated pseudo-images	Data heterogeneity, Privacy preservation

#### 4.3.1 Model aggregation methods

Model aggregation refers to the combination of multiple client updates into a global model update. One of the first proposed methods is Federated Stochastic Gradient Descent (FedSGD) [46], where clients compute gradients based on their local data and send these gradients to the server for aggregation at each iteration. Federated Averaging (FedAvg) [56] extends FedSGD by allowing clients to perform multiple local gradient updates before communicating the updated model weights with the central server. The averaging process can be weighted based on the size of each client's dataset, which ensures that clients with more data have a proportionally greater impact on the global model. FedAvg has become the standard algorithm for aggregating model updates in FL. However, as FedAvg does not address data heterogeneity (non-IID data), several advanced model aggregation techniques have been developed. Federated Proximal (FedProx) [48] adds a “proximal term” to the local objective function, which acts as a regularizer that penalizes large deviations from the global model. In simpler terms, it constrains the local updates, making them stay closer to the global model. In an alternative method, Stochastic Controlled Averaging in Federated Learning (SCAFFOLD) [45] uses local control variates at each client that estimate the global update direction and adjusts local updates to reduce the bias introduced by heterogeneous data. Another strategy for handling non-IID data is Personalized FL (PFL) [50], where each client keeps its own model version, by not updating a subset of private or “personal” layers. Notable PFL examples are FedBN [49], with personalized batch normalization (BN) layers, Local-Global FedAvg (LG-FedAvg) [51], which personalizes the base layers and keeps the top layers shared, conversely, FedPer [52] personalizes the top layers and keeps the base layers shared.

Further, three novel aggregation methods have been proposed targeting CPATH applications: Proportionally fair FL (Prop-FFL) [33] aims to overcome bias in client consideration by introducing a second loss objective that rewards a similar training loss across all clients, while also accounting for the proportion of training samples at each client. Clients with fewer samples receive a lower weight. Prop-FFL has been shown to reduce the standard deviation in accuracy across clients' held-out test sets by 5% for kidney and 11% for lung cancer subtyping compared to FedSGD. However, like FedSGD, Prop-FFL operates on gradients, requiring client-server communication after every batch. When extended to epoch-level communication, Prop-FFL is outperformed by FedAvg. Inspired by dropout in neural network training, Federated Dropout Averaging (FedDropoutAvg) [32] randomly drops a subset of model parameters from each client, or even completely drops client submissions, before aggregation. This approach serves as a regularization, enhancing model generalization while simultaneously preserving privacy, as not all model parameters are shared. In the pilot study [32] FedDropoutAvg outperformed FedAvg by 3% and 1% in F1-Score for colorectal tumor detection on a held-out test set and an external test set, respectively. SiloBN [31] is a PFL method that keeps the batch normalization (BN) parameters private for each client, excluding them from the model aggregation. This allows the global model to adapt to each local dataset, but requires computation of BN statistics for any unseen datasets during inference. In a held-out test set for breast tumor detection, SiloBN outperformed FedAvg by 1-2% in AUC.

#### 4.3.2 Model alignment methods

Three methods [38], [55], [39] introduce additional loss objectives during local training to align the global and client models: FL Barlow Twins [38] employs contrastive learning techniques by comparing tile-level representations learned by local and global models, ensuring that the cross-correlation matrix approximates the identity matrix. In [55], the feature embeddings from all clients are averaged for each class to create a “global prototype.” Local models are then trained to reduce the L2 distance between their embeddings and the global prototypes. For slide-level representation, [39] uses a Swin transformer for feature extraction and Attention MIL for feature aggregation, where an attention-consistency is imposed by adding Kullback-Leibler (KL) divergence between client and server model attention distributions to the loss function. This is to ensure that the regions of interest identified by the attention mechanism are consistent across different models, thus improving the image feature representation generation at the slide level. Both [38] and [39] enhance FedAvg by a modest improvement of 0∼3% in accuracy on held-out and external test sets for breast IDC grading and prostate tumor detection, respectively, indicating the limitation of relying solely on model alignment for improvement. A higher performance gain is notable in [55] with +2−4% accuracy in a monte-carlo cross validation for lung and +11% for kidney cancer subtyping. However, the missing validation on a held-out or external test set restricts the interpretability of the results with regards to model generalization. Beyond model alignment, we note that FedMM is the only method that enables clients without multi-modal data to participate in training and thereby provides them access to a multi-modal model.

#### 4.3.3 Domain alignment methods

We consider four studies [35], [36], [37], [38] that address specific CPATH data heterogeneity challenges, such as staining and scanning variations, through data domain alignment. In [35], [36], [37], a GAN is trained for federated stain normalization and applied to each client's data to generate more uniform stain appearance for downstream tasks. [35] proposes a cGAN with one global generator and multiple client-specific discriminators. Only the generator is shared and aggregated at the FL server after it is locally trained together with each client's discriminator. [36] extends the method by adding a temporal self-distillation objective to stabilize training, using an exponential moving average of successive global generator weights as the teacher model. [37] introduces a novel BottleGAN architecture which is first locally trained to perform two-way transform between a reference staining style and the client specific staining style. The client BottleGANs are then sent back to the server and applied to a reference dataset to generate images in multiple client staining styles. Finally, using the generated images, a global BottleGAN is trained on the server to normalize staining styles across all clients. [38] takes a different domain alignment approach by integrating self-supervised learning (SSL) as a pre-training step before FL. In this approach, pseudo images are generated by a multi-scale gradient GAN (MSG-GAN) [57] from each client, and these images are used to pre-train the backbone network using multi-task SSL. The pre-trained weights then serve as the parameter initialization for the downstream task. Overall, GAN-based stain normalization showed strong performance improvement (>10%) on tile- and pixel-level tasks, as well as slide-level tasks (4%) compared to no normalization. This highlights the need for sophisticated domain alignment methods in CPATH FL applications.

#### 4.3.4 Privacy preservation methods

Privacy preservation is a critical aspect of FL, as it ensures that sensitive data remains secure and confidential throughout the learning process. Several techniques have been developed to enhance privacy in FL. Here, we focus on Differential Privacy (DP) and Secure Multi-party Computation (SMC) as key CPATH use cases, which were investigated in four studies. DP [19] adds noise to the data or model updates to prevent the exposure of individual data points. A Gaussian noise generator is utilized with $N∼({\left(n\cdot \eta \right)}^{2})$, where z indicates the noise level and is the standard deviation of the respective model weight. SMC [58] ensures that the central server can only see aggregated updates from a cluster of N clients instead of individual updates, as they are masked by cryptographic techniques. A crucial aspect to this approach in FL is the security-performance trade-off, as a restriction in shared information often results in performance degradation. [39] implements DP with a noise factor of z=0.1 in FedAvg and FACL, resulting in equal performance with and without DP (±1% AUC) for tumor detection and grading. [41] investigates different noise factors (z∈{0,0.001,0.01,0.1,1.0}) on three different tasks, where z≤0.1 shows slight performance degradation (−2% AUC) for BRCA subtyping, RCC subtyping (±0.2% AUC) and CCRCC survival prediction (−4% c-Index), while z=1 results in significantly worse performance across all tasks (>15% reduction in AUC and c-index). Lastly, [42] explore differential private SGD, where gradient norms are clipped and noise is added to prevent information leakage. A norm clipping of 1.0 and a noise factor z=4 result in similar mean accuracy for lung cancer subtyping on a held-out test set, but a 4% accuracy drop on an external test set. [43] compares DP (z=0.03) to SMC (2 clusters of size N=3), showing SMC has less accuracy degradation (-0.5%) compared to DP (-6%) for FedAvg. In summary, all the proposed privacy methods show equal or slightly worse performance on CPATH tasks. The privacy loss is not measured by the majority of studies, only [42] quantifies the privacy loss with a privacy budget.

## 5 Editorial perspective: the road ahead

Utilizing FL enables us to capitalize on real-world hospital data, and develop algorithms that can potentially make better diagnostic decisions without divulging sensitive patient data. While FL is highly beneficial, it has noteworthy drawbacks compared to conventional centralized training methods. As in FL the data is distributed across clients, the optimization space is non-identically distributed which can result in slower convergence or reduced performance of the model. In [34], the FL training time was reported as twice as centralized training time. In addition, there is a cost associated with development and maintenance of communications in FL implementations. Considering both advantages and limitations of FL, we highlight the following CPATH use cases as particularly interesting for FL application. First, developing algorithms for rare cancers or cancer subtypes where the public dataset is sparse or non-existent. Although the data at each individual institution may not be sufficient for developing a robust model locally, through FL a model could be trained on the pool of data without the need for institutions to share sensitive patient data [44]. Second, FL can enable multi-institutional studies, such as large-scale epidemiological studies and multi-center clinical trials, which could strongly benefit from collaboration but under the restriction of protecting sensitive patient data. [59] demonstrates the feasibility of FL-enabled medical studies. Third, weakly-supervised learning and self-supervised learning (SSL). Given the fact that ground truth annotations in digital pathology are very expensive to obtain, and prone to variations across different annotators, weakly and self-supervised learning approaches are becoming increasingly popular in CPATH. These approaches are closer to real clinical applications, as slide level labels are generated more routinely than region or cell level labels during real clinical practice. On the other hand, training MIL and SSL based models usually requires huge datasets, immense computational power, and AI/ML expertise. Not all institutions have access to these resources, and as a result research exchange and validation of new methodology are limited to particular institutions with those capabilities. Combining FL and weakly or self-supervised learning approaches can empower all institutions, small or large, to combine their weakly labeled or unlabelled data, and computational resources to develop CPATH models. In [38], SSL and FL were successfully integrated for classification of histopathological images in a simulated environment. Lastly, FL systems allow rapid prototyping and evaluating for new algorithms on decentralized, private data. These systems are often easier to adapt to data privacy laws compared to conventional training and validation approaches that rely on centralized data and thereby enable algorithms to be more rigorously evaluated on otherwise inaccessible data.

The highlighted use cases demonstrate the potential of FL in CPATH, but as FL is still a relatively new concept, particularly in the field of medical imaging and CPATH, its implementation necessitates the development of additional guidelines and the promotion of standardized software frameworks. Most existing FL studies primarily focus on addressing data heterogeneity and privacy in simulated environments, while challenges related to establishing a real-world FL ecosystem, including system and hardware heterogeneity and the absence of a unified FL framework remain underexplored. This involves selecting from various open-source FL frameworks, such as NVFlare [60], Flower [61], PySyft [62], Tensorflow Federated [63], Substra [64] or FLAg [65], and requires network design expertise to establish a global server accessible to all clients, including opening ports inside restrictive hospital networks to enable client-server communication. Additionally, technical and programming expertise is necessary to adapt the chosen framework to specific applications. Unlike non-medical FL applications, the pathology setting typically involves fewer clients and larger datasets. Hence, it is critical to ensure that all clients contribute to the training process, even if they are slower than other clients or introduce heterogenous data. In summary, a practical guide for setting up real-world medical FL, including strategies to address common challenges such as data and system heterogeneity and privacy preservation, as well as advancements toward the standardization and unification of FL frameworks, is critical for transforming FL from a niche technology into a powerful tool for collaborative development in CPATH.

## 6 Conclusion

In this survey, we reviewed the technological advancements of federated learning (FL) in the context of computational pathology, addressing the unique challenges of this domain. Computational pathology deals with large image sizes requiring tiling of whole slide images and handling significant variations due to different tissue preparation, staining, and scanning processes. FL must address its inherent challenges — privacy, data heterogeneity, and system heterogeneity — while also managing the unique demands of computational pathology, with data heterogeneity being the most critical shared issue. Our review focused on advancements in four categories: model aggregation, model alignment, domain alignment, and privacy preservation. We found that while most technical advancements focus on aggregation and model alignment methods, their overall impact on test performance is relatively minor (0−3%). In contrast, domain alignment methods, though less prevalent, demonstrate a substantially higher impact (4−20%), underscoring the effectiveness of data-centric approaches. Privacy-preserving techniques maintain comparable performance while reducing the amount of shared information, although the quantification of privacy loss remains partial. Despite these advancements, considerable challenges persist, particularly regarding real-world implementation and scalability. The lack of standardized guidelines and frameworks complicates the development of large-scale FL solutions. Additionally, motivating clients to participate in FL and securing the necessary investment for FL infrastructure are unresolved issues. Addressing these concerns is vital for the broader adoption and success of federated learning in computational pathology.

## CRediT authorship contribution statement

**Lydia A. Schoenpflug:** Writing – review & editing, Writing – original draft, Visualization, Methodology, Conceptualization. **Yao Nie:** Writing – review & editing, Writing – original draft, Supervision, Project administration, Conceptualization. **Fahime Sheikhzadeh:** Writing – review & editing, Writing – original draft. **Viktor H. Koelzer:** Writing – review & editing, Supervision, Project administration.

## Declaration of Competing Interest

The authors declare the following financial interests/personal relationships which may be considered as potential competing interests: Yao Nie reports a relationship with F Hoffmann-La Roche Ltd that includes: employment and equity or stocks. Fahime Sheikhzadeh reports a relationship with F Hoffmann-La Roche Ltd that includes: employment and equity or stocks. Viktor H. Koelzer reports a relationship with 10.13039/100004337F. Hoffmann-La Roche Ltd that includes: funding grants. Viktor H. Koelzer reports a relationship with SPCC that includes: speaking and lecture fees. Viktor H. Koelzer reports a relationship with Indica Labs that includes: speaking and lecture fees. Viktor H. Koelzer reports a relationship with Takeda that includes: board membership. Viktor H. Koelzer reports a relationship with Swiss Digital Pathology Initiative that includes: board membership. Viktor H. Koelzer reports a relationship with IAG (Image Analysis Group) that includes: consulting or advisory. Lydia A. Schoenpflug reports a relationship with Indica Labs that includes: travel reimbursement. If there are other authors, they declare that they have no known competing financial interests or personal relationships that could have appeared to influence the work reported in this paper.
